# Reducing surgical site infection rates in colorectal surgery – a quality improvement approach to implementing a comprehensive bundle

**DOI:** 10.1111/codi.15875

**Published:** 2021-09-02

**Authors:** Rachel Falconer, George Ramsay, Jemma Hudson, Angus Watson, the Highland Colorectal SSI Group

**Affiliations:** ^1^ Department of General Surgery Raigmore Hospital Inverness UK; ^2^ Department of General Surgery Aberdeen Royal Infirmary, Aberdeen and Rowett Institute for Nutrition and Health, Foresterhill University of Aberdeen Aberdeen UK; ^3^ Health Services Research Unit University of Aberdeen Aberdeen UK

**Keywords:** bundles, colorectal surgery, surgical site infection, quality improvement

## Abstract

**Aim:**

Surgical site infections (SSIs) are a preventable cause of morbidity following surgical procedures. Strategies to reduce rates of SSI must address pre‐, peri‐ and postoperative factors and multiple interventions can be combined into ‘bundles’. Adoption of these measures can reduce SSIs, but this is dependent on high levels of compliance. The aim of this work is to assess the change in rates of SSI in elective colorectal surgery after implementing a colorectal SSI bundle.

**Method:**

This is a single‐centre prospective cohort study. All elective colorectal procedures from 2011 until 2018 (inclusive) were included. The primary outcome was inpatient SSI. A multimodal bundle was implemented using quality improvement methodology. The bundle was altered during the timeframe of the study to optimize outcomes. Data were analysed by interrupted time series analysis assessing points at which the bundle was altered.

**Results:**

In the study period, 1075 elective colorectal procedures were performed. Prior to the introduction of the colorectal SSI bundle, the SSI rate was 16.4%. During the implementation period (2013–2015), the overall rate of SSI fell from 15.9% to 9.4%, with the most significant reduction being in superficial SSI, from 8.6% to 4.7%. In the postimplementation period from 2015–2018, there was a further reduction in the overall rate of SSI (5.1%). In 2018, there were 87 consecutive cases without infection.

**Conclusion:**

A successful reduction in the rate of SSI following elective colorectal surgery can be achieved by adopting a comprehensive perioperative bundle. This is complemented by a process of continuous measurement and evaluation. The current bundle has achieved a significant reduction in superficial SSI.

**What does this paper add to the literature?**

Surgical site infections (SSIs) confer additional morbidity and mortality to elective colorectal surgery. We demonstrate that a coordinated multidisciplinary approach to implementing a ‘bundle’ of interventions can successfully reduce rates of SSI. We propose that adoption of this bundle is generalizable to other units and could act as a baseline for future work in this field.

## INTRODUCTION

The colon contains approximately 10^14^ live luminal bacteria, including multiple human pathogens [1]. Any operation in which the colon is opened is therefore a clean‐contaminated or a contaminated procedure, with an inherent risk of infection in the surrounding deep tissue or skin surface. The reported rates of surgical site infection (SSI) following colorectal surgery vary (often 10%–30% [2, 3, 4), but are consistently higher than the rates for other general surgical specialities [5]. However, high rates of SSI following colorectal surgery should not be accepted as inevitable or unchangeable.

SSIs are the most common cause of healthcare‐associated infections (HAIs) and confer an additional morbidity and mortality to the surgical procedure originally performed. SSIs can contribute to a prolonged hospital stay and increased readmission and intervention rates. They can delay rehabilitation and a return to normal activity, which may have a significant psychological impact [6]. Furthermore, SSIs confer an additional 3% risk of mortality after colorectal resection [3]. This is significant, given that colorectal resection is a common elective procedure in the UK (*n* = 18,796 in 2019) [7]. As a result, SSIs have a substantial economic burden, with an associated 35% increase in direct healthcare costs [8, 9.

Interventions to reduce rates of SSI in colorectal surgery are therefore needed to optimize both patient care and healthcare budgets. These measures must address a multitude of pre‐, intra‐ and postoperative risk factors. Adoption of groups of evidence‐based interventions, so called ‘bundles’, can be effective in reducing the rate of SSI. Both the National Institute for Health and Care and Excellence and World Health Organization (WHO) guidelines support a set of core interventions (judicious hair removal with clippers and administration of appropriate prophylactic antibiotics, as well as maintenance of normothermia and normoglycaemia perioperatively) which have reduced the rates of SSI across all surgical specialities [10, 11. However, considerable heterogeneity remains in the components of colorectal‐specific SSI bundles. Despite this heterogeneity, recent meta‐analyses have shown that, regardless of these variations, implementing a SSI bundle reduces the risk of infection following colorectal surgery by up to 40% [2, 3.

Our unit is a large district hospital serving the Highlands and Islands of Scotland, UK with an estimated population of 330,000. On average, 120–150 elective colorectal procedures are performed annually by full‐time colorectal surgeons in a dedicated theatre. Prospective surveillance of colorectal SSIs following elective surgery has been ongoing in our unit since 2011. An initial 6 month pilot study showed higher than expected rates of SSI in this patient cohort, prompting the creation of a multidisciplinary working group to translate emerging evidence on SSI bundles into a practical strategy to lower the rates in elective colorectal patients.

The aim of this paper is to describe the dynamic process of implementing a comprehensive perioperative colorectal SSI bundle using quality improvement methodology. We also aim to assess the resultant effect on SSI rates after bundle adoption in this single‐centre cohort study.

## METHOD

### Patient selection and data collection

This is a single‐centre prospective patient cohort and interrupted time series (ITS) study. The study period was from January 2011 to December 2018. The inclusion criterion was patients aged ≥16 years undergoing elective (planned) colorectal resection at Raigmore Hospital, Inverness, Scotland. Both open and laparoscopic operations were included. SSI was defined as infection of the index surgical procedure occurring in an inpatient, using the internationally recognized Scottish Patient Safety Programme (SPSP) definitions of superficial, deep and organ/space SSI [12]. The exclusion criteria were paediatric patients, noncolorectal procedures and patients undergoing emergency colorectal operations.

A hospital‐based surveillance system for colorectal SSIs was developed between February and June 2011. Data were initially collected using a paper proforma kept in the patient's medical notes and then transferred to a digital database. An online data collection tool was subsequently developed to allow accurate and timely recording of data within the operating and recovery areas. Postoperative data collection was carried out by the infection control team (independent of the colorectal team). All SSIs identified in an inpatient, readmissions and reinterventions within 30 days were recorded. However, because the mechanism of recording involved the secondary sector only, community events were not collected.

### Implementation of change

Before implementation of the bundle, individual clinician preference predominated in relation to aspects of the bundle used for each case. After implementation, the aim was that each aspect of the bundle be utilized for every case and compliance measured.

A colorectal SSI working group was created in December 2012 comprising colorectal and general surgeons, anaesthetists, microbiologists, scrub nurses, colorectal nurse specialists, ward nurses, hospital infection control team, patient safety and quality improvement advisors and hospital clinical governance advisors. Monthly meetings were held to address predicted issues in the bundle implementation. At these meetings, the constituent parts of the bundle were agreed by the group. Any changes to the bundle components were agreed by this group before they were implemented, based on how easy the interventions were to implement and anticipated compliance rates, in a pragmatic approach. A target of 95% compliance for each bundle component was agreed. Involvement from the Scottish Patient Safety Programme (SPSP) from October 2014 helped to create a standardized data collection tool.

Bundle implementation included education of ward and theatre staff to highlight the rationale for the change in practice and to help create a shared sense of responsibility for its development. There was dedicated teaching, which included face to face sessions as well as online modules to augment knowledge. Staff were actively encouraged to challenge nonadherence to the bundle where observed and to record any difficulties arising from factors such as lack of access to equipment. Relevant aspects of the bundle were incorporated into the patient safety brief and surgical pauses (sign in, time out and sign out) to help ensure compliance. Additional aids, such as a dedicated whiteboard and clock detailing antibiotic timing for the case, were installed in the colorectal theatre.

### Compliance and follow‐up

A robust evaluation of the bundle through continuous audit was undertaken. SSI incidence and bundle compliance were discussed monthly by the colorectal SSI group. Results were also disseminated electronically in the form of a monthly report. All recorded cases of SSI during the study period had a root cause analysis conducted to identify patient‐specific and system‐based issues. In instances where bundle compliance was less than 100%, justification for this was sought from key members of staff involved. Common themes, as well as specific issues which arose from these, were used as motivators for further improvement.

### Outcome measures

The primary outcomes were overall rates of SSI and rates of superficial SSI during the inpatient stay within secondary care. Readmissions and reinterventions were secondary outcomes.

### Statistical analysis

The primary aim of this study was to assess rate of overall, superficial and deep SSI over time in our unit. Generalized estimating equations (GEE) using segmented regression with a Poisson distribution using monthly data for the primary study outcome (in‐hospital SSI) were used. The model adjusted for covariates, gender, stoma creation, wound classification, type of surgery (open or laparoscopic), age (≤34, 35–54, 55–64, 65–74 and ≥75 years) and body mass index (BMI) (<19, 19–25, 25.1–30 and ≥30.1 kg/m^2^). If covariate data were missing, then a separate category was used. Due to sparse data, serial correlation was not accounted for. Effect sizes are either presented as incidence rate ratios with 95% confidence intervals (CIs) or converted to percentage change with 95% CIs.

**FIGURE 1:**
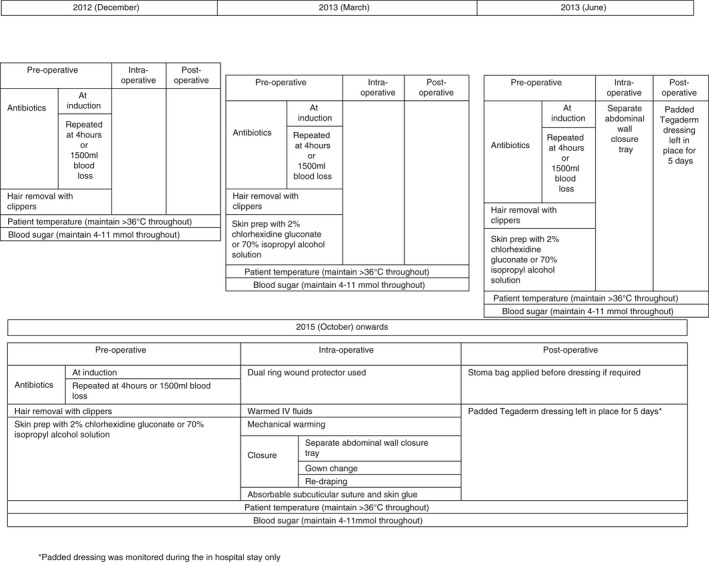
Changes to the colorectal surgical site infections (SSI) bundle over time (IV, intravenous)

There were four amendments to the colorectal SSI bundle implemented on 1 December 2012, 1 March 2013, 1 June 2013 and 1 October 2015 (Figure 1). A simple before and after assessment of treatment effect was not deemed appropriate due to the dynamic changes in the bundle over time. An ITS analysis was therefore undertaken. An ITS analysis constructs a time series of events for a particular event or outcome and is particularly helpful in the analysis of outcomes in quality improvement projects [13]. As the first three alterations happened within 7 months of each other these were modelled as one period. The model therefore had four periods: a preintervention period (February 2011 to November 2012); Period 1 (first postalteration period between December 2012 and May 2013); Period 2 (second postalteration period between June 2013 and September 2015); and Period 3 (third postalteration period between October 2015 and December 2018). The first month in each time period was not analysed to allow for the change to take effect. It was felt that a single month would be sufficient to implement each bundle change as the specific components of each change were relatively straightforward to undertake and were agreed upon by all stakeholders before their introduction. Results are summarized as preintervention period slope, change in level after each period, change in slope after each period and period slope. Figure S1 in the Supporting Information illustrates these effects. Demographic characteristics were also summarized by year and by period using appropriate summary statistics. All analysis was carried out in Stata 16 (Statacorp).

## RESULTS

A total of 1075 patients underwent an elective colorectal procedure between January 2011 and December 2018, of whom 228 had surgery in the preintervention period (January 2011–December 2012), 80 had surgery in Period 1 (December 2012–May 2013), 342 in Period 2 (June 2013–September 2015) and 386 in Period 3 (October 2015 to the end of the study).

Compliance data were continually collected and are shown in Figure S2. Figure 1 demonstrates changes to the components of the colorectal SSI bundle, from initial introduction in December 2012 to the final comprehensive bundle in October 2015.

Table 1 shows the demographic characteristics by time period (Table S1 shows demographics by year). Age, sex and BMI were similar across all periods, with the majority of patients classed as American Society of Anesthesiologists grade II (mild systemic disease). However, reporting of BMI was lost for the years 2017 and 2018. Most had open surgery in the preintervention period, Period 1 and 2, although in Period 3 the rate of laparoscopy increased to 41.5% of cases. Wound class was predominately clean‐contaminated across all periods. The mean length of hospital stay was between 9.8 and 10.6 days.

**TABLE 1:** Demographics by period

	Preintervention period (*N* = 228)	Period 1 (*N* = 80)	Period 2 (*N* = 343)	Period 3 (*N* = 386)
Age (years)	228; 65.0 (13.4)	80; 61.9 (15.3)	342; 63.6 (15.6)	386; 64.5 (15.4)
Sex				
Female	112 (49.1)	33 (41.2)	160 (46.8)	178 (46.1)
Male	116 (50.9)	47 (58.8)	182 (53.2)	208 (53.9)
BMI (kg/m^2^)	208; 27.4 (5.4)	80; 26.6 (5.7)	331; 26.6 (5.7)	383; 27.1 (5.9)
Missing BMI	20 (8.7)	0 (0)	11(3.2)	3 (0.7)
ASA grade				
Fit and well	27 (11.8)	15 (18.8)	50 (14.6)	18 (4.7)
Mild systemic disease	128 (56.1)	44 (55.0)	195 (57.0)	100 (25.9)
Severe systemic disease	65 (28.5)	20 (25.0)	90 (26.3)	43 (11.1)
Severe systemic disease, constant threat to life	8 (3.5)	1 (1.2)	7 (2.0)	–
Missing	–	–	–	225 (58.3)
Mode of surgery				
Open	174 (76.3)	66 (82.5)	267 (78.1)	226 (58.5)
Laparoscopic	54 (23.7)	14 (17.5)	73 (21.3)	160 (41.5)
Missing	–	–	2 (0.6)	–
Stoma created				
No	134 (58.8)	46 (57.5)	215 (62.9)	256 (66.3)
Yes	67 (29.4)	34 (42.5)	126 (36.8)	130 (33.7)
Unknown	27 (11.8)	–	1 (0.3)	–
Wound classification				
Clean‐contaminated	205 (89.9)	72 (90.0)	313 (91.5)	374 (96.9)
Dirty	17 (7.5)	5 (6.2)	25 (7.3)	9 (2.3)
Contaminated	6 (2.6)	3 (3.8)	4 (1.2)	3 (0.8)
Length of hospital stay (days)	197; 9.8 (6.6)	75; 9.6 (7.5)	329; 10.6 (8.5)	263; 10.5 (8.1)

Abbreviations

ASA, American Society of Anesthesiologists; BMI, body mass index.

Values are *n* (%) or *n*; mean (standard deviation). The preintervention period is 1 February 2011 to 30 November 2012; Period 1 (postintervention) is 1 December 2012 to 31 May 2013; Period 2 is 1 June 2013 to 30 September 2015; Period 3 is 1 October 2015 to 31 December.

**FIGURE 2:**
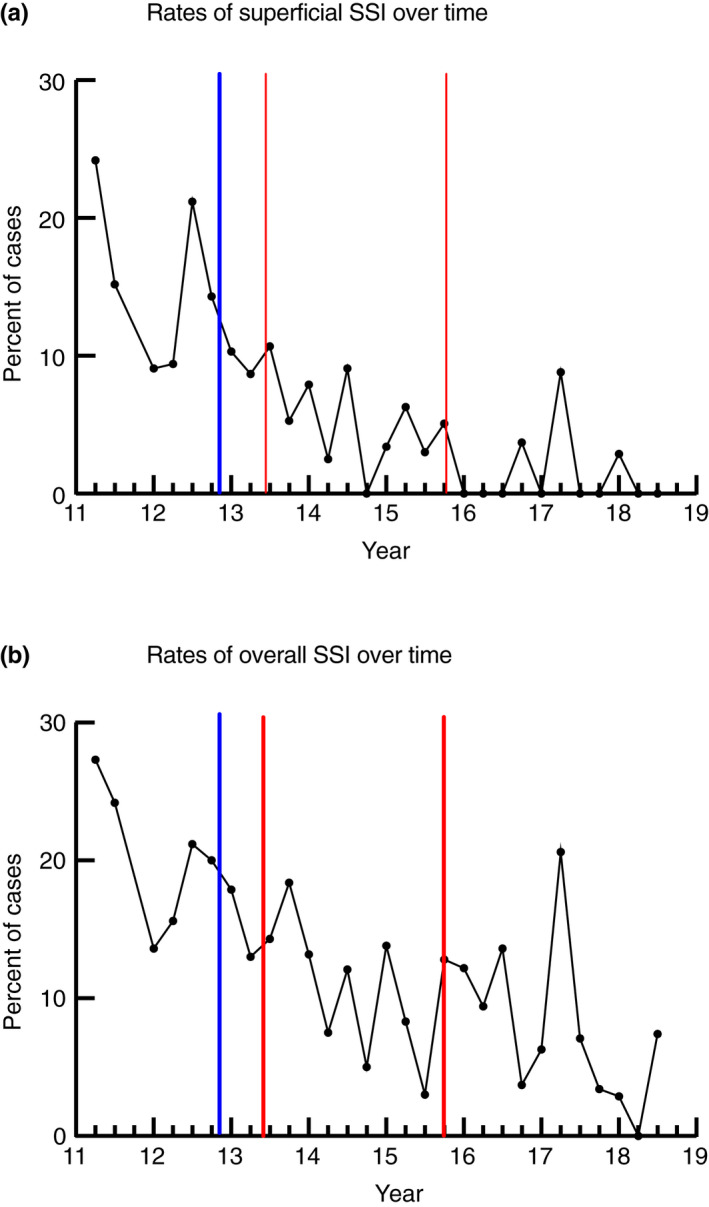
(A) Rates of superficial surgical site infections (SSIs) for patients who had surgery. (B) The total number of SSIs over time. Blue lines represent the start of the bundle implementation and the red lines indicate the timings of the change of components within the intervention

SSIs by period are shown in Table 2, with a further breakdown of SSI by year shown in Table S2. The number of SSIs in the preintervention period was 38 (16.7%) with 15 (18.8%) in Period 1. This decreased in Period 2 (35, 10.2%) and Period 3 (34, 8.8%). Overall, the SSI rate decreased from 16.4% in the first year of the study (2012) to 5.1% in the final year (2018) (Table S2). In the preintervention period and Periods 1 and 2 the majority of SSIs were superficial, but in Period 3, the type of SSI was predominately organ/space. Superficial SSI was identified in 2.3% of cases at the end of the study period. Figure 2(A) shows the number of superficial SSIs over time and Figure 2(B) shows the total number of SSIs over time.

**TABLE 2:** Surgical site infections (SSIs) by period

	Preintervention period (*N* = 228)	Period 1 (*N* = 80)	Period 2 (*N* = 342)	Period 3 (*N* = 386)
SSI present				
No	190 (83.3)	65 (81.3)	307 (89.8)	352 (91.2)
Yes	38 (16.7)	15 (18.8)	35 (10.2)	34 (8.8)

	Preintervention period (*N* = 38)	Period 1 (*N* = 15)	Period 2 (*N* = 35)	Period 3 (*N* = 34)
SSI type				
Superficial	30 (79.0)	10 (66.7)	17 (48.6)	9 (26.5)
Organ/space	7 (18.4)	5 (33.3)	15 (42.9)	20 (58.8)
Deep	1 (2.6)	‐	3 (8.6)	5 (14.7)
SSI by endoscopic status				
Open	35 (92.1)	11 (73.3)	28 (80.0)	26 (76.5)
Laparoscopic	3 (7.9)	4 (26.7)	7 (20.0)	8 (23.5)
Superficial and open SSI	28 (73.7)	7 (46.7)	16 (45.7)	8 (23.5)

Note

Values are *n* (%). The preintervention period is 1 February 2011 to 30 November 2012; Period 1 (postintervention) is 1 December 2012 to 31 May 2013; Period 2 is 1 June 2013 to 30 September 2015; Period 3 is 1 October 2015 to 31 December.

Incidence rate ratios from the segmented regression analysis for the number of superficial SSIs are shown in Table 3. This controlled for any changes in gender, stoma utilization, wound class, laparoscopic use, age and BMI. During the preintervention phase there was a rate of decrease of superficial SSI of 1% per month (95% CI −6% to 5%; *p*‐value 0.861). Immediately after the first intervention, there was an increase of 47% in the rate of SSI, with a 16% change in slope from the preintervention period to Period 1. For Period 1, there was a 16% decrease (95% CI −39% to 15%; *p*‐value 0.253) in the rate of SSI per month. There was a 14% change in slope from Period 1 to Period 2, with a 4% decrease in the rate of SSI per month in Period 2. Immediately after Period 2 (the implementation of the fourth intervention) there was an increase of 18% in the rate of SSI. There was a 3% change in slope from Period 2 to Period 3, with an increase of 19% (95% CI −13% to 63%; *p*‐value 0.267) per month in the rate of SSI in Period 3. There were similar results for total SSI (Table 3).

**TABLE 3:** Segmented regression analysis for superficial surgical site infections (SSIs) and total SSIs

	Incidence rate ratio	95% CI	*p*‐value
Superficial SSIs			
Preintervention slope	0.99	(0.94, 1.05)	0.861
Change in level from preintervention to Period 1	1.47	(0.42, 5.11)	0.542
Period 1 change in slope	0.84	(0.61, 1.15)	0.275
Period 1 slope	0.84	(0.61, 1.15)	0.253
Change in level from Period 1 to Period 2	1.72	(0.36, 8.09)	0.495
Period 2 change in slope	1.14	(0.83, 1.56)	0.421
Period 2 slope	0.96	(0.88, 1.04)	0.310
Change in level from Period 2 to Period 3	1.18	(0.20, 6.80)	0.277
Period 3 change in slope	1.03	(0.98, 1.08)	0.316
Period 3 slope	1.19	(0.87, 1.63)	0.267
Total SSIs			
Preintervention slope	0.99	(0.94, 1.05)	0.757
Change in level from preintervention to Period 1	1.61	(0.61, 4.23)	0.338
Period 1 change in slope	0.86	(0.68, 1.08)	0.199
Period 1 slope	0.86	(0.68, 1.08)	0.187
Change in level from Period 1 to Period 2	2.03	(0.70, 5.88)	0.191
Period 2 change in slope	1.11	(0.88, 1.40)	0.390
Period 2 slope	0.95	(0.98, 1.01)	0.122
Change in level from Period 2 to Period 3	3.15	(1.23, 8.06)	0.017
Period 3 change in slope	1.03	(0.98, 1.08)	0.316
Period 3 slope	1.14	(0.90, 1.42)	0.273

## DISCUSSION

In this prospective, single‐centre cohort study, we observed a significant reduction in SSIs in patients undergoing elective colorectal resection over time. The overall SSI rate decreased from 16.4% in the first year of the study to 5.1% in the last, following the implementation of a comprehensive SSI bundle. The most notable reduction was in the rate of superficial SSI (13.0% to 2.3%). Historically, colorectal surgery has been accepted as an outlier, with high SSI rates being justified by the nature of the surgery. Our results show that successful implementation of a colorectal SSI bundle appears to help achieve a reduction in postoperative SSIs equivalent to or even below the rates seen in other specialities. This approach was easy to adopt and our findings are likely to be generalizable to other units.

However, there is still debate about the optimal combination of bundle components. In colorectal surgery, preoperative warming measures have been described to be the single most effective intervention in reducing SSIs [14]. A subsequent meta‐analysis demonstrated significant benefit in reduction of SSI risk following colorectal surgery with several specific interventions including mechanical bowel preparation with oral antibiotics (55.4% vs. 31.8%, *p* = 0.015) as well as change of instruments and gloves prior to closure (58.6% vs. 28.5%, *P* = 0.019 and 56.9% vs. 28.5%, *p* = 0.002, respectively) [3]. However, a Cochrane review of evidence for intraoperative interventions in reducing SSIs across all specialities concluded there was limited evidence for any when analysed as individual interventions [15]. In this regard, the ROSSINI trial failed to demonstrate that use of wound edge protectors as a single intervention that could significantly reduce the rate of SSI following laparotomy [15]. However, subsequent meta‐analyses have demonstrated decreasing odds of SSI by their use [16]. In short, it is challenging to analyse individual interventions for reduction of SSI in the complex aetiology of wound infections in colorectal surgery.

A comprehensive approach to modifying risk factors for SSI appears to be required, with targeted pre‐, intra‐ and postoperative interventions. Comparable single‐centre studies show that the introduction of various colorectal SSI bundles can have a significant impact on the incidence of SSIs. In one Australian study of 408 patients, the crude SSI rate reduced from 15% to 7% following introduction of a multi‐intervention SSI bundle, despite variable compliance with individual bundle elements [4]. A larger study of 5120 from the USA with similar rates of open versus laparoscopic colorectal surgery was able to demonstrate a reduction in SSI rates from 9.8% to 4% following implementation of a multifactor SSI bundle [17]. These findings are corroborated by two recent meta‐analyses, comprising 8515 patients and 17,551 patients, respectively, which confirm a significant reduction in colorectal SSI rates after implementation of a dedicated bundle of care [2, 3. Our study adds to the literature by demonstrating the positive impact on infection rates of dynamic changes in a SSI bundle in line with changes in published best practice.

The evidence‐based bundles outlined above will have a limited effect without effective strategies for implementation. Compliance with SSI bundles in published studies is highly variable, ranging from 19% to 99% [3, 4, 17. Unsurprisingly, those with the lowest rates of overall compliance following bundle introduction often report little or no change in rates of SSI [14]. Compliance with individual bundle elements also has an additive effect in lowering the rate of SSI [17]. The staff commitment required to fully implement a successful bundle should not be underestimated. Indeed, it has been suggested that staff participation is such a vital factor that it should be incorporated in the SSI bundle as a specific intervention [14]. Each member of the team, from ward staff to theatre, needs to appreciate and feel valued in their role. Shared ownership of bundle interventions can help to move away from viewing SSIs as a purely ‘surgical problem’ [18]. By ensuring buy‐in and agreement by all stakeholders throughout this process, our compliance with each aspect of the bundle has remained high throughout.

One of the strengths of our approach was staff engagement during the process of bundle design, and implementation is crucial. A multidisciplinary work pattern appears to be most successful in reducing SSI rates [17, 19 and our experience supports this. In addition, systems of working which promote bundle compliance can be helpful. As in our study, Waits et al. demonstrated that integration of key bundle elements within the WHO surgical checklist is an effective strategy to aid compliance, leading to significantly lower rates of SSI [20, 21. Visual reminders (such as documentation of timings for subsequent antibiotic doses) was another simple strategy which we found to be effective. Limiting factors, such as a lack of forcing air warmers and other equipment, must be addressed at an organizational level if bundle implementation is to be successful [4].

Staff engagement is also key to building a sustainable system. Establishing the infrastructure to monitor the consistency of practice over time, as well as identifying developing issues, builds longer‐term success. Many studies demonstrate success in reducing SSI rates over 12 months or less, but our study provides more substantive longitudinal data. Documenting and sharing this success has been vital in maintaining staff motivation. In addition, collection of follow‐up data by an independent auditor or via an electronic system helps to maintain the integrity of the data [22]. Perhaps more importantly, rigorous interrogation of cases in which a SSI occurred or where compliance was poor is integral to improvement.

We acknowledge that this study has several limitations. Firstly, it is a single‐centre cohort study. However, the challenges faced in this work are likely to be familiar to most other units. We therefore think that this approach would be effective in other colorectal departments The HAI report published by NHS England highlighted that colorectal surgery had the biggest variability in SSI risk between participating hospitals [5]. Thus, sharing best practices from single centres helps to establish a framework for reduction of colorectal SSI nationally, allowing all patients to benefit.

Other limitations include a lack of information on comorbidity, smoking status or immunosuppressive medications or conditions, all of which can influence the rate of SSI. BMI was also not collected for the final 2 years of this study, which may have influenced some of the SSI rates. Length of operation was also not recorded in this work. Furthermore, our analysis only included elective patients. Although transferable to the emergency setting, this has not yet been universally established in our unit. Due to the methods of data collection, we were only able to analyse inpatient events, readmissions and reinterventions. This will therefore be an underestimation of the overall rate of SSI. However, we feel that the severe SSIs will be captured in this work and we had a consistent approach throughout this study. A further limitation is that we have not undertaken a cost analysis. Work to evaluate cost‐effectiveness is ongoing. We also acknowledge that the SSI trend was decreasing slightly before the start of this implementation, and therefore other factors not measured in this study may have influenced the improvements we observed. A further limitation is that only in‐hospital SSIs were identified in this assessment, missing those that will have been detected in the community.

Our experience shows that a significant and sustained reduction in postoperative infections, particularly superficial SSIs, can be achieved through effective and consistent implementation of a dedicated and comprehensive colorectal SSI bundle. A multidisciplinary approach in which all staff feel a shared responsibility for adhering to best practice is vital. A continuous cycle of evaluation is important when working towards optimizing both the bundle components and methods of delivery.

Colorectal units must therefore consider how best to translate the available evidence into a strategy to lower rates of postoperative SSI [23]. Successful implementation of these strategies relies on ‘buy‐in’ from the wider clinical team. In addition, a robust system of audit and evaluation is needed to ensure both consistent implementation and to facilitate critical appraisal when a SSI does occur. This allows the bundle to change and evolve over time, promoting further falls in SSI rates and achieving parity with those reported for many ‘cleaner’ specialities with traditionally lower rates of postoperative infection [5].

## CONFLICT OF INTERESTS

There are no conflicts of interest or anything else to disclose.

## ETHICAL APPROVAL

The study was approved by the NHS Highland clinical governance team. Formal ethics review was not deemed to be required.

## AUTHOR CONTRIBUTION

AJMW conceived the project and edited the manuscript. The NHS Highland SSI group co‐designed the programme and collated the data. JH performed statistical analysis and co‐authored the manuscript. RF performed data analysis and co‐authored the manuscript. GR performed data analysis and co‐authored the manuscript.

## Supporting information

Appendix S1

Figure S2

## Data Availability

Data available on request from the authors.
