# Supplementary material for: Reducing surgical site infection rates in colorectal surgery – a quality improvement approach to implementing a comprehensive bundle
Source: Colorectal Dis. 2021 Sep 2;23(11):2999–3007. doi: 10.1111/codi.15875 (PMC9293099; doi:10.1111/codi.15875)
Supplement: Supplementary file 1 — Appendix S1 [file CODI-23-2999-s001.docx]

**Supplementary Table 1. Demographics by year**

|  | **2011**  **N= 116** | **2012**  **N=122** | **2013**  **N=151** | **2014**  **N=151** | **2015**  **N=149** | **2016**  **N=122** | **2017**  **N=107** | **2018**  **N=118** |
| --- | --- | --- | --- | --- | --- | --- | --- | --- |
| Age (years) | 116; 65.1 (13.3) | 122; 64.3 (14.0) | 151; 63.6 (14.5) | 151; 62.8 (16.4) | 149; 63.5 (16.2) | 122; 62.9 (15.9) | 107; 65.0 (14.5) | 118; 66.2 (14.7) |
| Sex |  |  |  |  |  |  |  |  |
| Female | 57 (49.1) | 60 (49.2) | 63 (41.7) | 73 (48.3) | 68 (45.6) | 63 (51.6) | 47 (43.9) | 52 (44.1) |
| Male | 59 (50.9) | 62 (50.8) | 88 (58.3) | 78 (51.7) | 81 (54.4) | 59 (48.4) | 60 (56.1) | 66 (55.9) |
| BMI | 102; 27.7 (5.6) | 116; 27.1 (5.7) | 150; 27.0 (5.4) | 144; 26.9 (5.4) | 145; 26.9 (4.8) | 121; 26.4 (6.4) | 107; 27.7 (6.0) | 117; 27.3 (5.4) |
| ASA |  |  |  |  |  |  |  |  |
| Fit and well | 13 (11.2) | 15 (12.3) | 20 (13.2) | 28 (18.5) | 18 (12.1) | 16 (13.1) | - | - |
| Mild systemic disease | 68 (58.6) | 66 (54.1) | 86 (57.0) | 84 (55.6) | 92 (61.7) | 71 (58.2) | - | - |
| Severe systemic disease | 29 (25.0) | 39 (32.0) | 40 (26.5) | 37 (24.5) | 38 (25.5) | 35 (28.7) | - | - |
| Severe systemic disease, constant threat to life | 6 (5.2) | 2 (1.6) | 5 (3.3) | 2 (1.3) | 1 (0.7) | - | - | - |
| Missing | - | - | - | - | - | - | 107 (100.0) | 118 (100.0) |
| Mode of surgery |  |  |  |  |  |  |  |  |
| Open | 86 (74.1) | 97 (79.5) | 118 (78.1) | 119 (78.8) | 111 (74.5) | 60 (49.2) | 57 (53.3) | 85 (72.0) |
| Laparoscopic | 30 (25.9) | 25 (20.5) | 33 (21.9) | 30 (19.9) | 38 (25.5) | 62 (50.8) | 50 (46.7) | 33 (28.0) |
| Missing | - | - | - | 2 (1.3) | - | - | - | - |
| Stoma created |  |  |  |  |  |  |  |  |
| No | 59 (50.9) | 81 (66.4) | 93 (61.6) | 98 (64.9) | 90 (60.4) | 82 (67.2) | 68 (63.6) | 80 (67.8) |
| Yes | 30 (25.9) | 41 (33.6) | 58 (38.4) | 52 (34.4) | 59 (39.6) | 40 (32.8) | 39 (36.4) | 38 (32.2) |
| Unknown | 27 (23.3) | - | - | 1 (0.7) | - | - | - | - |
| Wound classification |  |  |  |  |  |  |  |  |
| Clean contaminated | 102 (87.9) | 111 (91.0) | 138 (91.4) | 143 (94.7) | 135 (90.6) | 119 (97.5) | 103 (96.3) | 113 (95.8) |
| Dirty | 12 (10.3) | 7 (5.7) | 9 (6.0) | 7 (4.6) | 12 (8.1) | 3 (2.5) | 3 (2.8) | 3 (2.5) |
| Contaminated | 2 (1.7) | 4 (3.3) | 4 (2.6) | 1 (0.7) | 2 (1.3) | - | 1 (0.9) | 2 (1.7) |
| Length of hospital stay | 93; 9.9 (6.3) | 112; 9.6 (6.6) | 144; 10.1 (7.1) | 147; 9.9 (5.2) | 144; 11.3 (11.7) | 0; . (.) | 106; 10.6 (7.5) | 118; 10.5 (8.4) |

Values are n (%) or n; mean (standard deviation)

**Supplementary Table 2. Surgical site infections by year**

|  | **2011**  **N= 116** | **2012**  **N=122** | **2013**  **N=151** | **2014**  **N=151** | **2015**  **N=149** | **2016**  **N=122** | **2017**  **N=107** | **2018**  **N=118** |
| --- | --- | --- | --- | --- | --- | --- | --- | --- |
| SSI |  |  |  |  |  |  |  |  |
| No | 97 (83.6) | 100 (82.0) | 127 (84.1) | 137 (90.7) | 135 (90.6) | 110 (90.2) | 96 (89.7) | 112 (94.9) |
| Yes | 19 (16.4) | 22 (18.0) | 24 (15.9) | 14 (9.3) | 14 (9.4) | 12 (9.8) | 11 (10.3) | 6 (5.1) |
|  | N=19 | N=22 | N=24 | N=14 | N=14 | N=12 | N=11 | N=6 |
| SSI Type |  |  |  |  |  |  |  |  |
| Superficial | 15 (78.9) | 17 (77.3) | 13 (54.2) | 7 (50.0) | 7 (50.0) | 1 (8.3) | 3 (27.3) | 3 (50.0) |
| Organ/Space | 4 (21.1) | 4 (18.2) | 10 (41.7) | 5 (35.7) | 7 (50.0) | 8 (66.7) | 8 (72.7) | 1 (16.7) |
| Deep | - | 1 (4.5) | 1 (4.2) | 2 (14.3) | - | 3 (25.0) | - | 2 (33.3) |
| SSI by Endoscopic |  |  |  |  |  |  |  |  |
| Open | 17 (89.5) | 20 (90.9) | 17 (70.8) | 13 (92.9) | 9 (64.3) | 9 (75.0) | 9 (81.8) | 6 (100.0) |
| Laparoscopic | 2 (10.5) | 2 (9.0) | 7 (29.2) | 1 (7.0) | 5 (35.7) | 3 (25.0) | 2 (18.2) | 0 (0.0) |
| Superficial and Open SSI | 14 (73.7) | 16 (72.7) | 9 (35.5) | 7 (50.0) | 6 (42.9) | 1 (8.3) | 3 (27.3) | 3 (50.0) |

Values are n (%)

**Supplementary Figure 1. Illustration of interrupted time series effect estimates.**

Change in slope is the difference between pre-slope and post-slope

Pre-slope

Intervention

Post-slope
