# Supplementary material for: Reducing surgical site infection rates in colorectal surgery – a quality improvement approach to implementing a comprehensive bundle
Source: Colorectal Dis. 2021 Sep 2;23(11):2999–3007. doi: 10.1111/codi.15875 (PMC9293099; doi:10.1111/codi.15875)

Supplementary figure 2: Compliance data runs.

Line delineates median compliance. Overall compliance indicates compliance with every aspect of the bundle (as it was designed at that time).


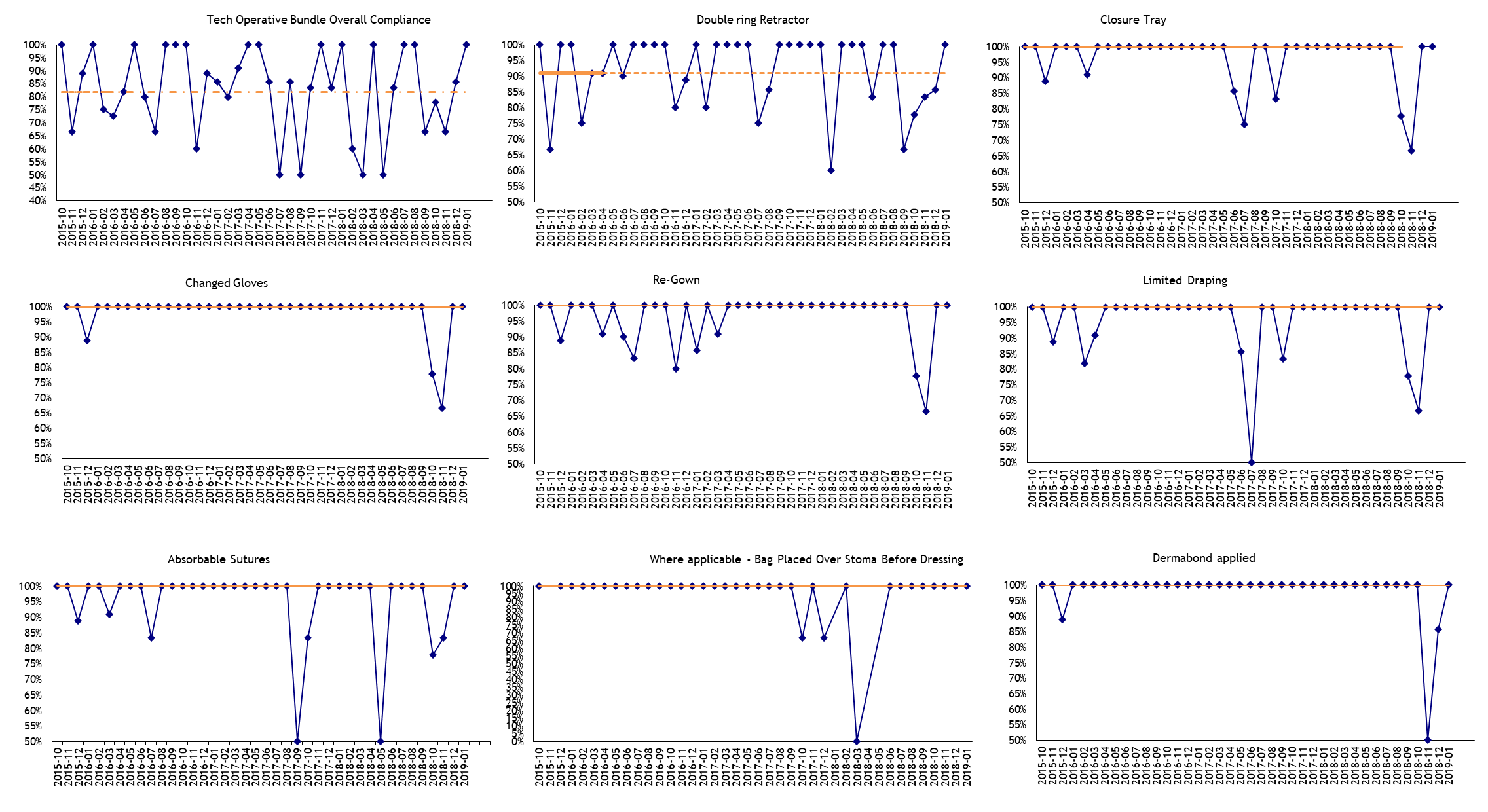


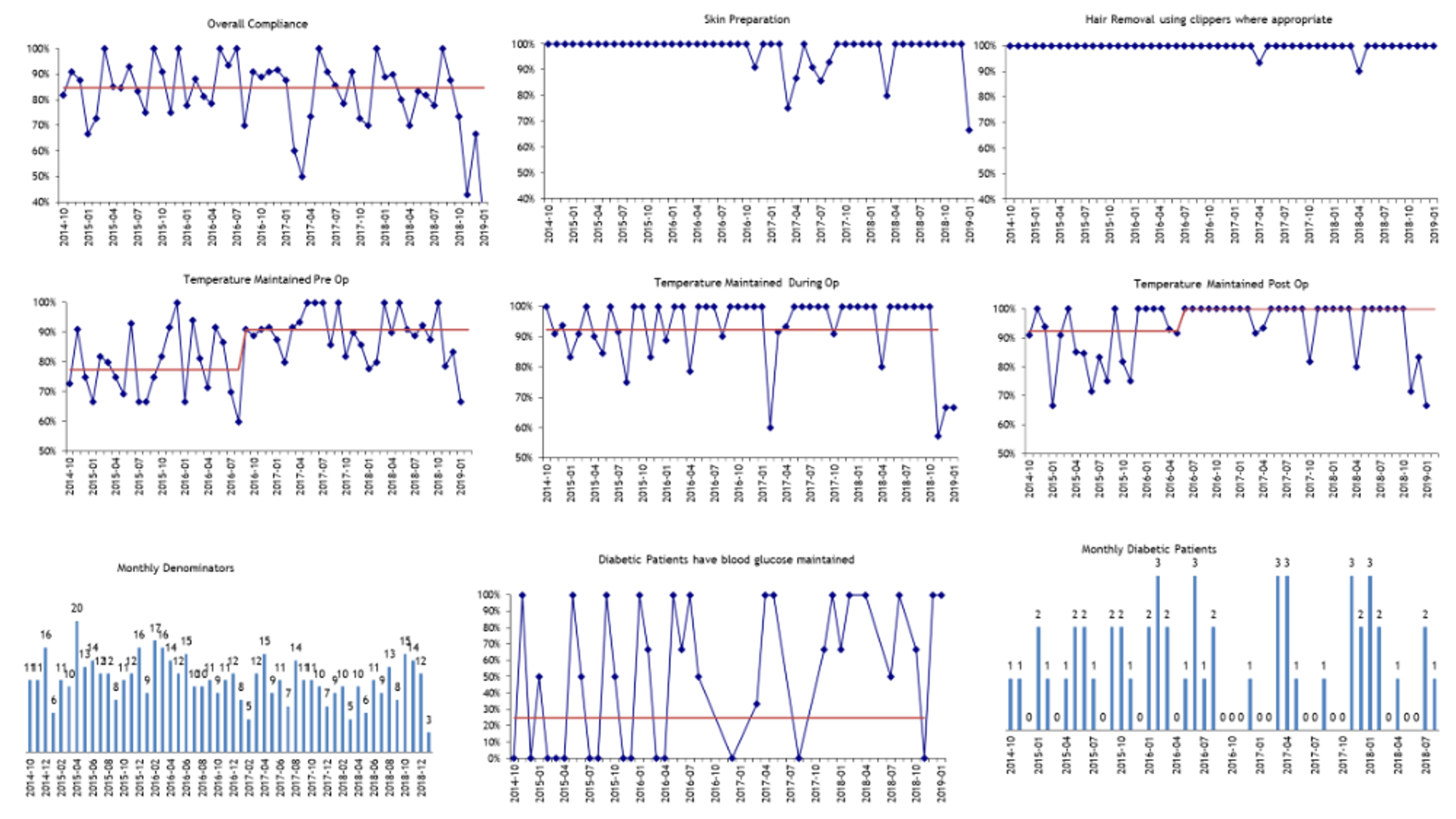

Supplement: Supplementary file 2 — Figure S2 [file CODI-23-2999-s002.docx]
